# A Gut Microbial Metabolite HYA Ameliorates Adipocyte Hypertrophy by Activating AMP-Activated Protein Kinase

**DOI:** 10.3390/nu17081393

**Published:** 2025-04-21

**Authors:** Rino Matsushita, Kaori Sato, Kunitoshi Uchida, Yukiko Imi, Reina Amano, Nanaho Kasahara, Yuki Kitao, Yuki Oishi, Honoka Kawaai, Chiaki Tomimoto, Yusei Hosokawa, Shigenobu Kishino, Jun Ogawa, Tetsuya Hosooka

**Affiliations:** 1 Laboratory of Nutritional Physiology, Graduate School of Integrated Pharmaceutical and Nutritional Sciences, University of Shizuoka, Suruga-ku, Shizuoka 422-8526, Japans24222@u-shizuoka-ken.ac.jp (Y.O.);; 2 Laboratory of Functional Physiology, Graduate School of Integrated Pharmaceutical and Nutritional Sciences, University of Shizuoka, Suruga-ku, Shizuoka 422-8526, Japan; 3 Noster Inc., Muko-shi 617-0006, Japan; 4 Division of Diabetes and Endocrinology, Department of Internal Medicine, Kobe University Graduate School of Medicine, Kobe 650-0017, Japan; yusei0719@yahoo.co.jp; 5 Division of Applied Life Sciences, Graduate School of Agriculture, Kyoto University, Sakyo-ku, Kyoto 606-8502, Japan; kishino.shigenobu.3e@kyoto-u.ac.jp (S.K.);

**Keywords:** HYA, AMPK, adipocytes

## Abstract

**Background/Objectives:** Metabolites produced by gut microbiota play an important role in the crosstalk between the gut and other organs. Although HYA (10-hydroxy-*cis*-12-octadecenoic acid), a linoleic acid metabolite produced by lactic acid bacteria represented by *Lactobacillus*, has been shown to exert physiological effects such as metabolic improvement and anti-inflammation in the host, its direct action on adipose tissue and the mechanism remains unknown. **Methods**: The effect of HYA administration on adipocyte size in mice fed a high-fat diet was examined. In 3T3-L1 mature adipocytes treated with HYA, the amount of intracellular lipid droplets was evaluated by Oil red O staining, gene expression by real-time qPCR, phosphorylation of AMP-activated protein kinase (AMPK) by immunoblotting, and intracellular Ca^2+^ concentration with calcium imaging. **Results**: Administration of HYA, but not linoleic acid, to obese mice fed a high-fat diet significantly reduced adipocyte size. To investigate whether the inhibition of adipocyte hypertrophy by HYA has a direct effect on adipocytes, 3T3-L1 adipocytes were treated with HYA, which significantly decreased the amount of intracellular lipid droplets in these cells. Gene expression analysis by real-time PCR showed decreased expression of genes related to lipogenesis such as FAS and ACC1, and increased expression of CPT1A, which is involved in fatty acid oxidation. Mechanistically, HYA was found to activate AMPK in adipocytes by increasing intracellular Ca^2+^ concentration. **Conclusions**: HYA suppresses adipocyte hypertrophy by activating AMPK in adipocytes. HYA may be a potential therapeutic for obesity and related metabolic disorders.

## 1. Introduction

Obesity is exploding worldwide and has become a major medical and social challenge. Obesity induces systemic insulin resistance, which leads to metabolic diseases including type 2 diabetes, dyslipidemia, hypertension, and cardiovascular diseases [1,2,3]. Recent studies have also shown that obesity is associated with an increased risk of many types of cancer [4,5]. Obesity is caused by changes in energy balance resulting from overnutrition and lack of exercise in addition to genetic factors. Although diet and exercise therapy are important for improving this pathological condition, it is often difficult to achieve sufficient weight loss and to maintain the weight loss for a long period of time through diet and exercise therapy. As the obese population is expected to continue to increase worldwide, it is necessary to develop new preventive and therapeutic methods for addressing obesity.

Adipose tissue plays a pivotal role in the regulation of systemic metabolism by regulating inter-organ crosstalk between adipose tissue and other organs through energy delivery in response to changes in energy status, such as fasting and feeding, and by regulating the production of adipokines including adiponectin and leptin [6,7]. On the other hand, a number of studies have shown that overnutrition and lack of exercise induce dynamic changes in adipose tissue, such as adipocyte hypertrophy followed by infiltration of inflammatory cells represented by macrophages, an increase in inflammatory cytokines/chemokines and decrease in adiponectin, and insulin resistance in adipocytes [8,9,10,11]. These changes in adipose tissue cause dysfunction of this tissue, which leads to the development of metabolic disorders. Since adipocyte hypertrophy is the initial trigger for adipose tissue dysfunction, the regulation of adipocyte hypertrophy may contribute to the development of preventive and therapeutic strategies for obesity and obesity-related metabolic diseases.

Recent studies have revealed that metabolites produced by gut microbiota play an important role in the crosstalk between the gut and other organs [12,13,14]. 10-hydroxy-*cis*-12-octadecenoic acid (HYA) is produced when linoleic acid is hydrated by the enzyme CLA-HY, which is possessed by lactic acid bacteria represented by *Lactobacillus* [15]. Given that the amount of linoleic acid metabolites, including HYA, is extremely low in the tissues of germ-free mice compared to specific pathogen-free mice, these metabolites are thought to be produced mainly by gut microbiota [15]. HYA exerts anti-inflammatory effects, such as improvement of intestinal epithelial barrier damage in dextran sulfate sodium-induced mouse colitis and amelioration of atopic dermatitis in NC/nga mice [16,17]. HYA has also been shown to suppress obesity in mice fed a high-fat diet and to improve glucose metabolism through GPR40- or GPR120-mediated GLP-1 secretion [18]. Furthermore, Nanthirudjanar et al. showed that HYA reduces lipid accumulation in hepatocytes [19], and we have recently reported that HYA ameliorates liver fibrosis in a mouse model of metabolic dysfunction-associated steatohepatitis (MASH) by inhibiting the TGF-β pathway in hepatic stellate cells [20]. Thus, HYA exerts its effects through several mechanisms, including anti-inflammatory, anti-obesity, and metabolism improvement. However, the direct effects of HYA on adipocytes are unknown. The aim of the present study is to investigate the direct effects of HYA on adipocytes and the mechanism underlying these effects.

## 2. Materials and Methods

### 2.1. Animal Study

Male 5-week-old C57BL/6J mice were obtained from CLEA Japan (Tokyo, Japan) and fed standard chow (MF, Oriental Yeast, Tokyo, Japan) for 5 days to acclimate. To generate an obese model, mice were fed a high-fat diet (HFD32, CLEA, Tokyo, Japan) for 6 weeks. Subsequently, mice were divided into three groups and fed a high-fat diet supplemented or not with 1% HYA (Noster Inc., Kyoto, Japan) or LA for an additional 5 weeks (*n* = 10 mice per group). During the experimental period, mice had free access to tap water and food, and were housed at 21° to 25 °C under a 12 h light/12 h dark cycle in the animal facility at University of Shizuoka and Kobe University Graduate School of Medicine. After 5 weeks, mice were euthanized under 2% isoflurane (VIATRIS, Tokyo, Japan) anesthesia (airflow, 1.5 L/ min), and then epididymal white adipose tissues were rapidly collected. All animal experiments were approved by and conducted in accordance with the animal ethics committees of University of Shizuoka (No. 215326) and Kobe University Graduate School of Medicine (No. P171006).

### 2.2. Histological Analysis

The tissue was fixed with 10% neutral buffered formalin and embedded in paraffin. The tissue sections were stained with hematoxylin and eosin (H&E). H&E images were captured using a BZ-X800 microscope (KEYENCE, Osaka, Japan) at 100× magnification. At least three fields of view were randomly selected from the tissue specimen of each mouse and the size of all adipocytes in the field of view was evaluated using BZ-X800 analyzer software v1.1.2.4 (KEYENCE, Osaka, Japan).

### 2.3. Cell Culture

3T3-L1 pre-adipocytes were maintained in maintenance medium (high-glucose Dulbecco’s modified Eagle’s medium (DMEM) containing 10% fetal bovine serum, 100 units/mL penicillin, and 100 μg/mL streptomycin) at 37 °C in a humidified atmosphere of 5% CO_2_. To induce differentiation into mature adipocytes, 100% confluent cells were maintained for 2 days before being switched to differentiation medium (maintenance medium supplemented with 10 μg/mL insulin, 1 μM dexamethasone, and 500 μM 3-isobutyl-1-methylxanthine). After 2 days, the medium was replaced with maintenance medium containing 10 μg/mL insulin for an additional 2 days. Subsequently, the medium was replaced with fresh maintenance medium every other day for an additional 4 days. Cells were used for the experiments 8 days after induction of differentiation. All experiments with 3T3-L1 cells were repeated at least three times to confirm technical reproducibility.

### 2.4. Oil Red O Staining

Differentiated 3T3-L1 adipocytes treated with HYA or LA (final concentration 30 or 100 μM) for 48 h were stained with Oil red O as described previously [21]. Briefly, the cells were washed with PBS and fixed with 10% neutral buffered formalin for 1 h at room temperature. After two washes with PBS, the cells were stained with the Oil red O solution in 60% isopropanol for 7 min. The stained adipocytes were washed with PBS and observed under a microscope. Oil red O was quantified using BZ-X800 analyzer software (KEYENCE, Osaka, Japan).

### 2.5. RT-qPCR Analysis

Total RNA was extracted from adipocytes treated with HYA or LA (final concentration 30 or 100 μM) for 48 h using the RNeasy Lipid Tissue Mini Kit (Qiagen, Venlo, The Netherlands). For reverse transcription (RT) and quantitative polymerase chain reaction (qPCR) analysis, the isolated RNA was subjected to RT using Prime Script RT Master Mix (Takara Bio, Shiga, Japan) and the resulting cDNA was subjected to qPCR analysis using TB Green Premix Ex Taq II (Takara Bio, Shiga, Japan) in a Thermal Cycler Dice Real Time System III (Takara Bio, Shiga, Japan). Relative mRNA abundance was determined by the standard curve method and normalized to the amount of *Rplp0* mRNA. The sequences of the PCR primers are provided in Table 1.

### 2.6. Immunoblot Analysis

Immunoblot analysis was performed as described previously [21]. In brief, cells were lysed in lysis buffer (20 mM Tris-HCl [pH 7.5], 150 mM NaCl, 2 mM EDTA, 1% Nonidet P-40, 10% glycerol) supplemented with protease and phosphatase inhibitors. The lysates were subjected to SDS-polyacrylamide gel electrophoresis, and the separated proteins were transferred onto a nitrocellulose membrane. For immunoblot analysis, the membrane was blocked with 5% skimmed milk for 1 h at room temperature and incubated overnight at 4 °C with antibodies to total (#5832, 5000× dilution) or phosphorylated forms (#2535, 5000× dilution) of AMPK to total (#3676, 5000× dilution) or phosphorylated forms (#11818, 5000× dilution) of ACC1 (Cell Signaling Technology, Beverly, MA, USA), and to GAPDH (#60004-1-Ig, 5000× dilution, Proteintech, Tokyo, Japan). The membrane was incubated with an appropriate secondary antibody (Promega, Madison, WI, USA, 2500× dilution) for 1 h at room temperature and visualized using FUSION SOLOS Imaging System (Vilber Bio Imaging, Collégien, France).

### 2.7. Calcium Imaging

3T3-L1 adipocytes were differentiated on micro coverslips in a 35 mm dish. The calcium imaging experiment was performed as described previously [22]. Briefly, cells were incubated with 3.3 μM Fura-2 AM (Thermo Fisher Scientific, Waltham, MA, USA) for at least 40 min. After incubation, the coverslips were placed in an open chamber (Warner Instruments LLC, Hamden, CT, USA) and perfused with standard bath solution (140 mM NaCl, 5 mM KCl, 2 mM CaCl_2_, 2 mM MgCl_2_, 10 mM glucose, and 10 mM HEPES, pH 7.4 adjusted with NaOH) for 3 min. Then, the coverslips were perfused with standard bath solution containing 0.01% DMSO, 300 μM HYA, or 300 μM LA for 10 min. Images of perfused cells were captured every 5 s using a CoolSNAP ES CCD camera (Photometrics, Tucson, AZ, USA). All experiments were conducted at 35° to 36.5 °C. Intracellular free Ca^2+^ concentrations in 3T3-L1 cells were measured by dual-wavelength Fura-2 microfluorometry with excitation at 340/380 nm and emission at 510 nm. The images were acquired using NIS Elements software v4.50 (NIKON Corp., Tokyo, Japan), and the ratio was calculated using ImageJ v1.52q (NIH, Bethesda, MD, USA). The viability of cells was confirmed using 3 μM ionomycin.

### 2.8. Statistical Analysis

Quantitative data are presented as means ± SEM. Group comparisons were performed using a two-tailed unpaired Student’s *t*-test or analysis of variance (ANOVA) followed by Tukey’s post hoc test, as appropriate. A *p* value of <0.05 was considered statistically significant.

## 3. Result

### 3.1. HYA Inhibits Adipocyte Hypertrophy in Mice Fed a High-Fat Diet

**Figure 1:**
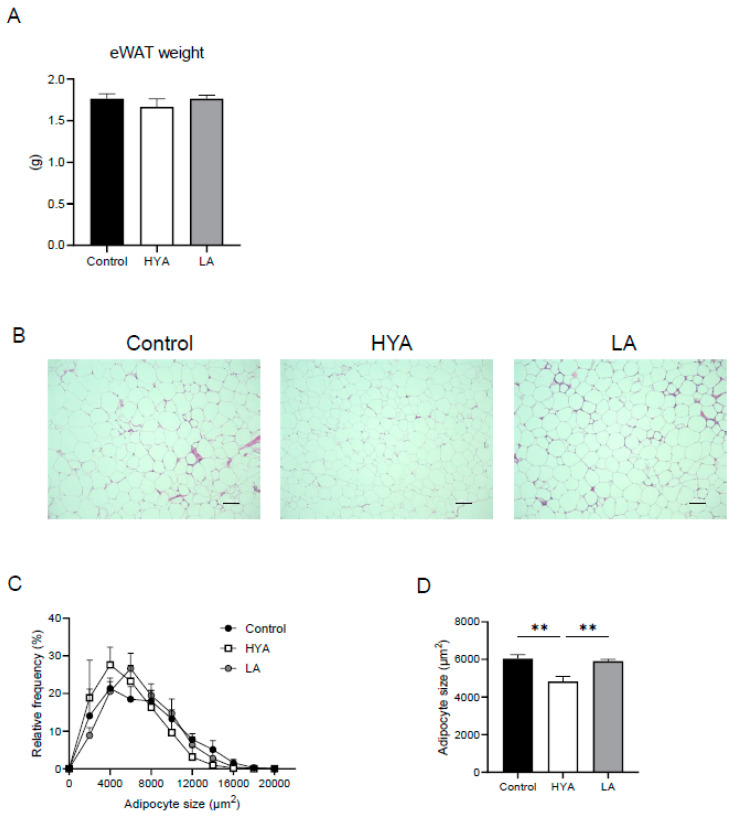
Effect of HYA treatment on adipocyte hypertrophy in mice fed a high-fat diet. (**A**) Weight of epididymal adipose tissue (eWAT) in mice fed a high-fat diet (Control) or a high-fat diet containing 1% HYA or 1% LA for 5 weeks after a 6-week high-fat diet (*n* = 10 mice per group). (**B**) Representative hematoxylin–eosin (H&E) staining for eWAT of mice as in (**A**). Original magnification, 100×. Scale bars, 100 μm. (**C**,**D**) Distribution of adipocyte size (**C**) and average of adipocyte size (**D**) shown in (**B**). Data in (**A**,**D**) are means ± SEM. ** *p* < 0.01 (one-way ANOVA followed by Tukey’s test).

To investigate the effects of HYA on adipose tissue, male C57BL/6J mice in which obesity was induced by a 6-week high-fat diet were divided into three groups, each receiving a high-fat diet (CT), a high-fat diet containing 1% HYA (HYA), or a high-fat diet containing 1% linoleic acid (LA) for another 5 weeks. Although no significant changes in fat mass were observed in mice fed HYA (Figure 1A), histological analysis of epididymal adipose tissue showed that adipocyte size was significantly reduced in HYA-fed mice compared to CT-fed mice. In contrast, no such effect was observed in LA-fed mice (Figure 1B–D).

### 3.2. HYA Decreases the Amount of Lipid Content in Cultured Adipocytes

**Figure 2:**
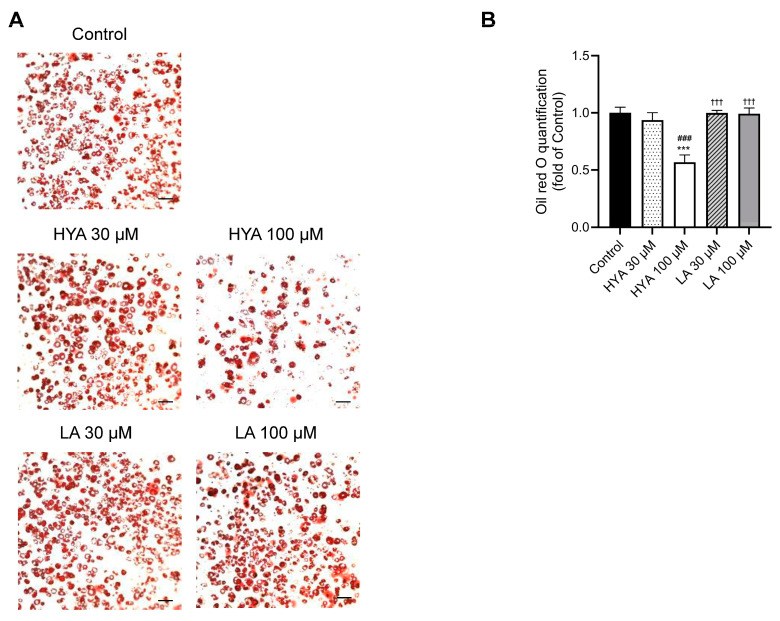

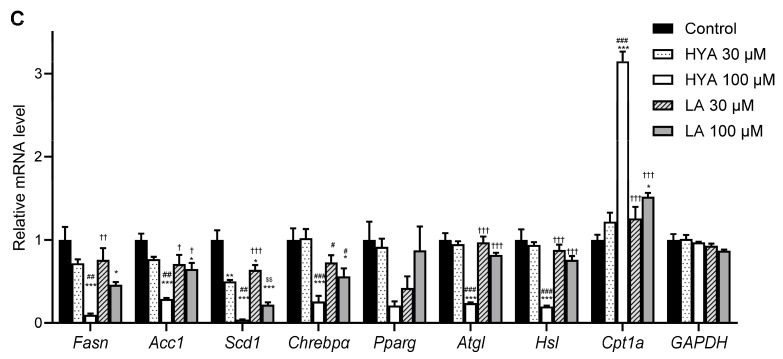
Effect of HYA on lipid content in 3T3-L1 adipocytes. (**A**) Representative Oil red O staining of 3T3-L1 adipocytes incubated in the absence (Control) or presence of HYA (10 or 30 μM) or LA (10 or 30 μM) for 48 h. Original magnification, ×100. Scale bars, 100 μm. (**B**) Quantification of Oil red O staining shown in (**A**). (**C**) RT-qPCR analysis of mRNA abundance for lipid metabolism-related genes in 3T3-L1 adipocytes treated under the same conditions as in (**A**). Data in (**B**,**C**) are means ± SEM (*n* = 3). * *p* < 0.05, ** *p* < 0.01, *** *p* < 0.001 versus control; # *p* < 0.05, ## *p* < 0.01, ### *p* < 0.001 vs. HYA 30 µM; † *p* < 0.05, †† *p* < 0.01, ††† *p* < 0.001 versus HYA 100 µM; $$ *p* < 0.01 versus LA 30 µM (one-way ANOVA followed by Tukey’s test).

To investigate whether the effect of HYA in vivo has a direct effect on adipocytes, we performed experiments using cultured adipocytes. 3T3L1 cells were differentiated into mature adipocytes. After treatment of 3T3-L1 differentiated adipocytes with HYA, intracellular lipid content was evaluated by Oil Red staining. HYA-treated 3T3-L1 differentiated cells showed significantly reduced staining by Oil Red compared to untreated 3T3-L1 differentiated cells (Figure 2A,B). Consistent with the results of Oil Red staining, gene expression analysis by real-time PCR showed that the expression of genes related to lipogenesis such as FAS, ACC1, SCD1, and ChREBPα decreased in a HYA concentration-dependent manner (Figure 2C). PPARγ expression also showed a decreasing trend, although it did not reach statistical significance (Figure 2C). Furthermore, the expression of CPT1A, which is involved in fatty acid utilization, was increased by HYA treatment (Figure 2C). The expression of genes related to lipolysis including ATGL and HSL was decreased by HYA treatment. The effect of LA on such gene expression was relatively small compared to HYA. These results suggest that HYA suppresses adipocyte hypertrophy by inhibiting lipogenesis and promoting fatty acid oxidation.

### 3.3. HYA Activates AMP Kinase in Adipocytes

**Figure 3:**
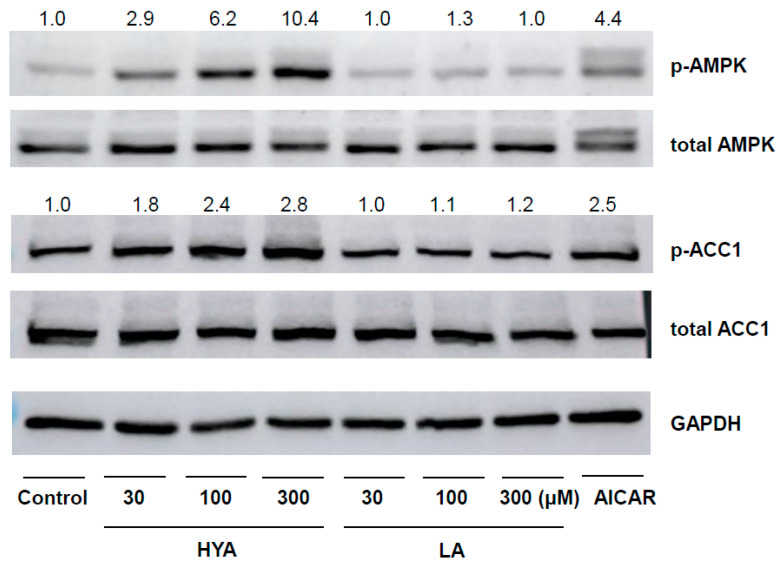
Effect of HYA on AMPK activation in 3T3-L1 adipocytes. Immunoblot analysis of total and phosphorylated (p) forms of AMPK and ACC1, and GAPDH in 3T3-L1 adipocytes incubated in the absence (Control) or presence of HYA (30, 100, or 300 μM), LA (30, 100, or 300 μM), or 1 mM AICAR for 1 h. The numbers at the top of the lane indicate the extent of the phosphorylation form to total protein as expressed relative to Control.

We focused on AMPK as the molecular mechanism of HYA action in differentiated adipocytes, a kinase that acts as a cellular energy sensor and plays an important role in metabolic regulation in response to changes in energy status [23,24]. Immunoblotting using phosphorylated antibodies of AMPK showed that HYA increased phosphorylation of AMPK in a concentration-dependent manner in 3T3-L1 differentiated adipocytes (Figure 3). Consistent with the increased phosphorylation of AMPK, HYA enhanced downstream ACC1 phosphorylation (Figure 3). In contrast, LA treatment did not induce such AMPK phosphorylation. These results suggest that the activation of AMPK by HYA is involved in the reduction of lipid content in adipocytes.

### 3.4. HYA Activates AMPK by Increasing Intracellular Ca^2+^ Concentration

**Figure 4:**
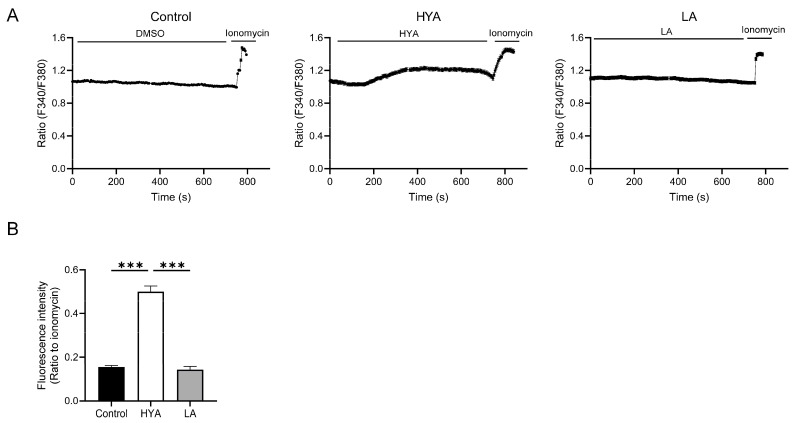
Effect of HYA on intracellular Ca^2+^ concentration in 3T3-L1 adipocytes. (**A**) Changes in intracellular Ca^2+^ concentrations in 3T3-L1 adipocytes incubated with DMSO (Control), HYA (300 μM), or LA (300 μM). *Y*-axis: Fura-2 ratio (340/380 nm). (**B**) Ratio of the peak response during treatment with DMSO, HYA, or LA to that of ionomycin treatment in (**A**). Data in (**B**) are means ± SEM. *** *p* < 0.001 (one-way ANOVA followed by Tukey’s test).

One of the mechanisms of AMPK activation involves an increase in intracellular Ca^2+^ concentration [24,25]. To clarify the mechanism of AMPK activation by HYA, the effect of HYA on intracellular Ca^2+^ concentration in adipocytes was examined by Ca^2+^ imaging using a Fura2-AM probe. Compared to HYA-untreated 3T3-L1 differentiated cells, intracellular Ca²^+^ concentrations were significantly increased in HYA-treated 3T3-L1 differentiated adipocytes (Figure 4A,B). In contrast, no change in intracellular Ca^2+^ concentration was observed in 3T3L1 differentiated cells treated with LA (Figure 4A,B). These results suggest that HYA activates AMPK by increasing intracellular Ca^2+^ concentration.

### 3.5. HYA Reduces Lipid Content in Adipocytes by a Mechanism Independent of GPR40 and GPR120

**Figure 5:**
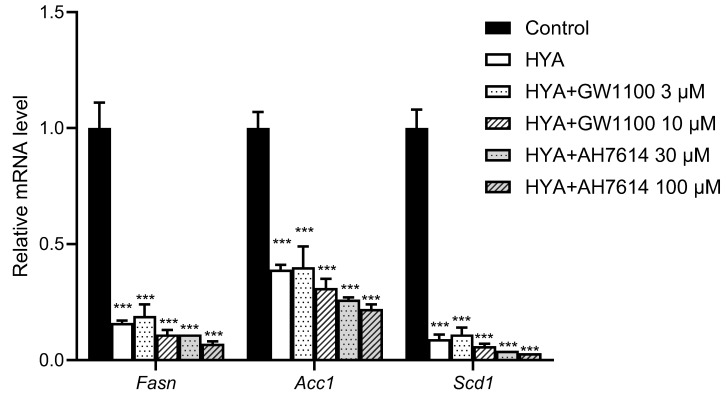
Effect of inhibitors for GPR40 or GPR120 on HYA-mediated suppression of gene expression related to lipogenesis in 3T3-L1 adipocytes. RT-qPCR analysis of mRNA abundance for lipogenesis-related genes in 3T3-L1 adipocytes treated in the absence (Control) or presence of HYA (100 μM) and inhibitors for GPR40 (GW1100) or for GPR120 (AH7614) for 48 h. Data are means ± SEM (*n* = 3). *** *p* < 0.001 versus control (one-way ANOVA followed by Tukey’s test).

Long-chain fatty acids, including HYA, have been reported to act on GPR40 and GPR120 as their receptors [18,26]. To determine whether the action of HYA in adipocytes is mediated by GPR40 and GPR120, we examined the effects of GPR40 or GPR120 inhibitors on the HYA-induced decrease in the expression of lipogenic genes in 3T3-L1 differentiated adipocytes. Real-time PCR analysis showed that HYA again suppressed the expression of lipogenic genes including Fas, Acc1, and Scd1, whereas treatment with GPR40 or GPR120 inhibitors did not cancel the HYA-induced decrease in the expression of these lipogenic genes (Figure 5). These results suggest that HYA acts in adipocytes by a mechanism independent of GPR40 and GPR120.

## 4. Discussion

Recent studies have revealed that metabolites produced by gut microbiota play an important role in the crosstalk between the gut and other organs [12,13,14]. HYA, a metabolite produced by lactic acid bacteria such as *Lactobacillus*, has been reported to have several physiological functions in the host, but its direct action on adipocytes and the mechanism remained unclear. In this study, we demonstrated for the first time that HYA suppresses adipocyte hypertrophy through AMPK activation. Thus, our study adds a new mechanism for metabolic regulation by organ crosstalk between the gut and other tissues.

HYA has been reported to have anti-inflammatory effects, including improvement of intestinal epithelial barrier damage in dextran sulfate sodium-induced mouse colitis and atopic dermatitis in NC/nga mice [16,17]. HYA has also been reported to improve systemic metabolism. Miyamoto et al. showed that HYA improves glucose metabolism by promoting GLP-1 secretion via GPR40 and GPR120 in L cells [18]. They also reported that HYA reduces adipocyte size in mice fed a high-fat diet [18], consistent with our results. Although these effects of HYA have been shown to be accompanied by a decrease in food intake [18], we have now shown a mechanism by which HYA acts directly on adipocytes. Thus, HYA may act to suppress adipocyte hypertrophy through its direct action on adipocytes in addition to the effects on the central nervous system including feeding regulation. Given that adipocyte hypertrophy triggers adipose tissue dysfunction and subsequent systemic metabolic disorders, HYA may have a favorable effect on systemic metabolism, at least in part through its action on adipocytes. We have recently shown that HYA ameliorates liver fibrosis in a MASH model by suppressing TGF-β signaling in hepatic stellate cells [20]. HYA has also been reported to reduce TG levels in hepatocytes by suppressing SREBP-1c expression [19]. Although it is not determined whether HYA activates AMPK in the liver, the ameliorative effect of HYA on MASH may involve a reduction in fat accumulation through AMPK activation in the liver.

It is known that the regulatory mechanism of AMPK activity involves an increase in intracellular Ca^2+^ concentration [24,25]. In the present study, we found that HYA increases intracellular Ca^2+^ concentration in cultured adipocytes. The mechanism by which HYA increases intracellular Ca^2+^ concentrations in adipocytes was not determined in this study. Although HYA has been reported to activate the G protein-coupled receptors GPR40 and GPR120 in cultured L cells [18], pharmacological inhibition of both receptors did not cancel the effect of HYA in cultured adipocytes, suggesting that HYA regulates intracellular Ca^2+^ concentrations by a mechanism independent of GPR40 and GPR120 in adipocytes. There may be another GPCR that mediates the action of HYA. Another possibility is that a TRPV1-mediated mechanism may be involved. A previous paper showed that KetoA, a metabolite produced from HYA in the process of linoleic acid saturation, enhances energy metabolism by activating TRPV1 [27]. Given that TRPV1 is expressed predominantly in sensory neurons but also peripheral tissues including adipose tissue [28], it is possible that HYA may increase intracellular Ca^2+^ concentration in adipocytes via TRPV1. Another possible mechanism is that HYA is taken up by adipocytes and directly affects certain signals and transcription factors. In addition to intracellular Ca^2+^ concentration, the AMP/ATP ratio regulates AMPK activity [23,24]. Therefore, future studies are needed to determine whether HYA affects the AMP/ATP ratio in cells including adipocytes.

The aryl hydrocarbon receptor (AhR) is a ligand-activated transcription factor that has been implicated in organ-crosstalk by gut microbial metabolites. In addition to its well-known roles as a xenobiotic sensor, recent studies have shown that AhR plays important roles in a variety of physiological and pathophysiological conditions [29]. There are a number of endogenous and exogenous AhR ligands, the former including gut microbial metabolites such as indole and kynurenic acid [30,31]. Recent studies have reported that the activation of AhR by indole metabolites generated by gut microbiota is involved in the mechanism of crosstalk between the gut and other organs [32]. Activation of AhR in adipocytes has been reported to suppress adipocyte differentiation by down-regulating PPARγ expression [33,34]. PPARγ is known to play key roles in adipocyte differentiation as well as in the regulation of lipogenesis in mature adipocytes [35]. Since PPARγ expression showed a decreasing trend with HYA treatment in 3T3L1 differentiated adipocytes in the present study, the possible involvement of AhR in the action of HYA as well as the direct action of HYA on PPARγ in adipocytes needs to be investigated in the future. The AhR-PPARγ axis in adipose tissue might be involved in the mechanism of crosstalk between the gut and adipose tissue by gut microbial metabolites including HYA.

Although the action of AMPK on lipolysis and its mechanism have not been fully elucidated compared to its action on lipogenesis in adipocytes, it has been proposed that AMPK activation suppresses cAMP-dependent lipolysis [36]. Two lipases, HSL and ATGL, play important roles in lipolysis in adipocytes, and it has been shown that AMPK phosphorylates the serine 565 residue of HSL to inhibit its activity as a mechanism of AMPK-dependent inhibition of lipolysis [37]. We found that HYA suppressed the expression of ATGL and HSL in this study, while it is unknown whether AMPK is involved in the regulation of expression of these lipases. Therefore, future studies will be required to determine whether the HYA-dependent decrease in HSL and ATGL expression is mediated by AMPK activation or through another mechanism. In addition, whether HYA induces HSL phosphorylation in adipocytes also needs to be investigated in future studies. Although the effect of HYA on lipolysis was not investigated in this study, HYA treatment leads to a decrease in the amount of lipid droplets in adipocytes, suggesting that HYA may have a predominant inhibitory effect on lipogenesis rather than lipolysis in adipocytes.

## 5. Conclusions

In this study, we show for the first time that HYA inhibits adipocyte hypertrophy by activating AMPK. Adipocyte hypertrophy plays an important role in the development of adipose tissue dysfunction and the subsequent systemic metabolic disorders. The mechanisms of metabolic regulation by HYA uncovered in this study provide an important basis for the development of new therapies for obesity and obesity-associated metabolic disorders.

## Figures and Tables

**Table 1:** The sequences of the PCR primers.

Gene	Forward Primer (5′→3′)	Reverse Primer (5′→3′)
*Fasn*	GCTGGCATTCGTGATGGAGTCGT	AGGCCACCAGTGATGATGTAACTCT
*Acc1*	GATGAACCATCTCCGTTGGC	GACCCAATTATGAATCGGGAGTG
*Scd1*	TTCTTGCGATACACTCTGGTGC	CGGGATTGAATGTTCTTGTCGT
*Chrebp*α	CACCTCTTCGAGTGCTTGAG	CATAGCAACTTGAGGCCTTTG
*Pparg*	GGGGATGTCTCACAATGCCA	CAGACTCTGGGTTCAGCTGG
*Atgl*	GCCAACGCCACTCACATCTA	AATGTTGGCACCTGCTTCAC
*Hsl*	TATGGAGTGAGGGTGCCAGA	ATGGTCCTCTGCCTCTGTCC
*Cpt1a*	GCTGCTTCCCCTCACAAGTTCC	GCTTTGGCTGCCTGTGTCAGTATGC
*Gapdh*	AACTTTGGCATTGTGGAAGG	GGATGCAGGGATGATGTTCT
*Rplp0*	GAGGAATCAGATGAGGATATGGGA	AAGCAGGCTGACTTGGTTGC

## Data Availability

The original contributions presented in the study are included in the article, further inquiries can be directed to the corresponding author.

## Abbreviations

ACC1	acetyl-CoA carboxylase 1
AhR	aryl hydrocarbon receptor
ATGL	adipose triglyceride lipase
ATP	adenosine triphosphate
AMP	adenosine monophosphate
CLA-HY	conjugated linoleic acid hydrase
ChREBPα	carbohydrate-responsive element-binding protein α
CPT1A	carnitine acyltransferase 1A
FAS	fatty acid synthase
GLP-1	glucagon-like peptide-1
GPCR	G protein-coupled receptor
GPR40	G protein-coupled receptor 40
GPR120	G protein-coupled receptor 120
HSL	hormone-sensitive lipase
HYA	10-hydroxy-*cis*-12-octadecenoic acid
LA	linoleic acid
MASH	metabolic dysfunction-associated steatohepatitis
PPARγ	peroxisome proliferator-activated receptor gamma
SCD1	stearoyl-CoA desaturase 1
TGF-β	transforming growth factor-β
TRPV1	transient receptor potential vanilloid 1
